# Supplementary material for: Evidence supported by Mendelian randomization: impact on inflammatory factors in knee osteoarthritis
Source: Front Med (Lausanne). 2024 May 28;11:1382836. doi: 10.3389/fmed.2024.1382836 (PMC11165061; doi:10.3389/fmed.2024.1382836)
Supplement: Supplementary file 1 [file Data_Sheet_1.ZIP › data/GCST90274791_5e6/791.funnel_plot.pdf]

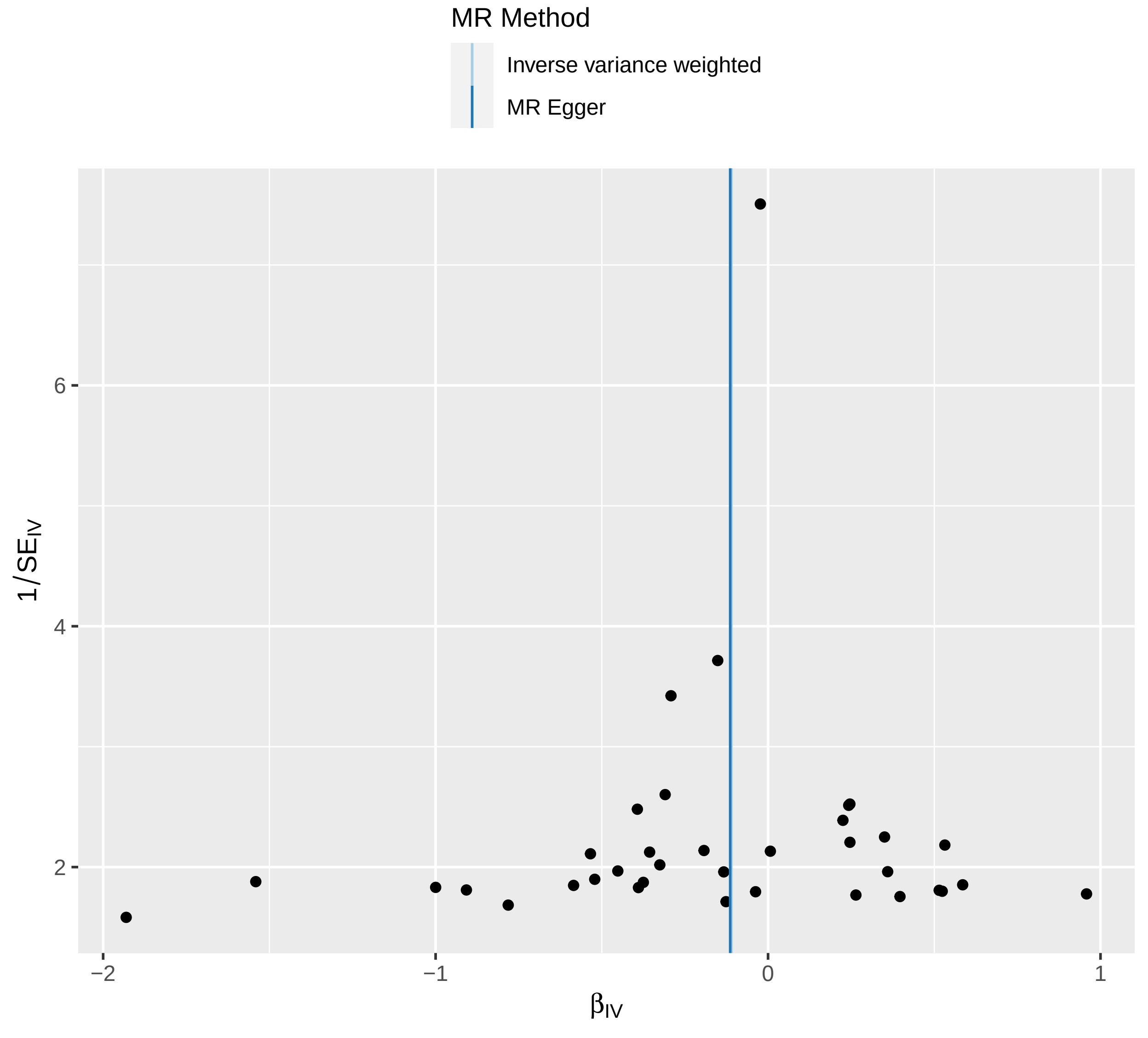

MR Method
Inverse variance weighted
MR Egger
1/SE_IV
6
4
2
−2
−1
0
1
β_IV
